# Development of Antioxidant-Active Sericin–Curcumin-Loaded Sodium Alginate/Polyvinyl Alcohol Films Crosslinked with Calcium Chloride as a Promising Wound Dressing Application

**DOI:** 10.3390/polym16223197

**Published:** 2024-11-18

**Authors:** Rungnapha Yamdech, Vareesa Terahsongkran, Varis Terahsongkran, Sarocha Cherdchom, Pornanong Aramwit

**Affiliations:** 1 Department of Pharmacy Practice, Faculty of Pharmaceutical Sciences and Center of Excellence in Bioactive Resources for Innovative Clinical Applications, Chulalongkorn University, Bangkok 10330, Thailand; rungnapha.yamdech@gmail.com; 2 Mater Dei School, 534 Phloen Chit Rd., Lumphini, Pathum Wan, Bangkok 10330, Thailand; vareesa.terahsongkran@gmail.com; 3 Patumwan Demonstration School, Srinakharinwirot University, Henri Dunant Rd., Pathum Wan, Bangkok 10330, Thailand; varis.gusto@gmail.com; 4 Department of Preventive and Social Medicine and Center of Excellence in Nanomedicine, Faculty of Medicine, Chulalongkorn University, Bangkok 10330, Thailand; sarocha.cherdchom@gmail.com; 5 The Academy of Science, The Royal Society of Thailand, Dusit, Bangkok 10330, Thailand; 6 Faculty of Pharmacy, Silpakorn University, Nakhon Pathom 73000, Thailand

**Keywords:** sericin, curcumin, sodium alginate/polyvinyl alcohol (SA/PVA) films, calcium chloride crosslinking, wound healing

## Abstract

Silk sericin (SS) and curcumin (Cur) possess significant antioxidant properties, making them highly beneficial for wound healing applications. This study aimed to develop SS–Cur-loaded sodium alginate/polyvinyl alcohol (SA/PVA) films crosslinked with calcium chloride, creating a biomaterial with enhanced stability and antioxidant properties. Wound dressings containing SS-Cur were fabricated by mixing SA and PVA at different ratios of 1:1, 1:2, 1:4, and 1:6. The resulting films were then crosslinked with calcium chloride in an ethanol solution to enhance film integrity. These films were characterized using several techniques, revealing that the presence of ethanol in calcium chloride affected film properties, including the gel fraction, swelling, film thickness, and FTIR analysis. The presence of ethanol in calcium chloride revealed the highest drug content in the SA/PVA films. In vitro release studies demonstrated sustained release of SS-Cur from all formulations. Cytotoxicity and antioxidant activity tests showed that SS–Cur-loaded SA/PVA films with ethanol in calcium chloride increased cell viability and enhanced antioxidant effects in L929 cells. In conclusion, this study demonstrates that the presence of ethanol in the crosslinking solution improved the functionality of SS–Cur-loaded SA/PVA films, making them promising candidates for wound healing and soft tissue regeneration.

## 1. Introduction

Physical damage to the skin, such as cuts, abrasions, trauma, and burns, disrupts the body’s largest organ and exposes underlying tissues to potential infections [1]. The process of wound healing restores the integrity of the skin, but complications such as chronic wounds can arise, particularly in individuals with underlying health conditions [2,3]. The development of novel wound dressing materials using biomaterials has emerged as a promising approach to enhancing the healing process [4]. These advanced dressings are designed to provide optimal conditions for healing by maintaining a moist environment, protecting against infections, or delivering therapeutic agents directly to the wound site [4,5]. The integration of biomaterials into wound dressings represents a significant advancement in medical technology, offering potential improvements in the treatment and management of various types of wounds.

Moist dressings, such as thin films, hydrogels, foams, hydrocolloids, and composites, play a critical role in wound healing by maintaining a moist environment at the wound site [6]. Among these, hydrogels are particularly promising due to their ability to regulate moisture levels, promote gas exchange, and absorb excess exudates effectively [6]. However, some traditional options have drawbacks: hydrocolloid dressings may lead to irritant or allergic contact dermatitis in certain patients [7,8], while foam dressings have been associated with malodorous discharge similar to the odor from hydrocolloids [8]. As a result, hydrocolloid and foam dressings are generally not recommended for infected wounds or those with heavy drainage [9].

Special attention has been given to sodium alginate (SA) and polyvinyl alcohol (PVA). Alginate, a natural polysaccharide derived from brown seaweed, offers exceptional benefits in wound care due to its high absorbency, biocompatibility, and ability to form hydrogels [5,10,11,12,13]. PVA, a synthetic polymer known for its film-forming properties, mechanical strength, and flexibility, complements alginate by enhancing the structural integrity and durability of the wound dressing [12,14,15,16]. Unlike hydrocolloid and foam dressings, SA/PVA films are highly compatible with bioactive compound loading, enabling controlled, sustained drug release [6]. Together, alginate and PVA create a synergistic effect, resulting in advanced dressings that effectively manage wound exudates, promote a conducive healing environment, and provide a barrier against microbial invasion. This innovative combination holds great promise for improving the efficacy of wound care.

Antioxidants play a pivotal role in wound healing by neutralizing reactive oxygen species (ROS), which are highly reactive molecules that can cause significant cellular damage [17]. Incorporating ROS-scavenging compounds into wound dressings significantly enhances the healing process of skin wounds [18,19]. Drug-incorporating biomaterials, such as antioxidant compounds, provide antioxidant protection and offer additional benefits, such as anti-inflammatory and antimicrobial effects, to provide a valuable opportunity in wound application [18,20,21,22]. Wound dressings composed of silk sericin (SS) and curcumin (Cur) are considered alternative topical wound healing materials. The antioxidant effects of SS and Cur play a crucial role in wound healing by neutralizing harmful free radicals and reducing oxidative stress, which can otherwise impede the healing process. SS, a protein derived from the silkworm *Bombyx mori*, is recognized for its excellent biocompatibility and notable antioxidant properties due to its ability to scavenge free radicals and protect cells from oxidative damage, making it a valuable component in biomedical applications [23]. Similarly, Cur, the active compound found in turmeric, is renowned for its antimicrobial properties and powerful antioxidant activity, which helps to mitigate oxidative stress and inflammation [24,25]. The synergistic effect of sericin and curcumin has been explored in both in vitro and in vivo studies, demonstrating their combined effectiveness in inflammatory conditions [26]. The combination of SS and Cur was most effective in reducing IL-1β levels and inducing IL-4 and IL-10 expression in lipopolysaccharide (LPS)-stimulated McCoy cells compared to SS or Cur alone [26]. Additionally, the combined treatment significantly reduced paw thickness and circumference in mouse models of hind paw edema, indicating enhanced anti-inflammatory effects over SS or Cur treatment alone [26]. Wound dressings that incorporate SS and Cur represent an innovative approach to wound care, offering a dual-function system that supports rapid and effective wound healing while providing a protective barrier against infection and further injury.

The objective of this research was to develop SS–Cur-loaded SA/PVA films by crosslinking with calcium chloride in an ethanol solution at various ratios to achieve optimal formulations. The physicochemical properties of the films were investigated, including film morphology, gel formation, swelling behavior, and FTIR analysis. Additionally, the antioxidant capacities and release characteristics of SS and Cur were measured. This study aims to provide valuable insights into the development of advanced wound dressings that combine the beneficial properties of SS and Cur, offering enhanced film stability, controlled release, and antioxidant protection for improved wound healing outcomes.

## 2. Materials and Methods

### 2.1. Preparation of Films

#### 2.1.1. Synthesis of SS–Cur-Loaded SA/PVA Films

In the first step, polyvinyl alcohol (PVA) (degree of polymerization 1700–1800, hydrolysis 98–99%, 0531500500, Labochemie, Jehangir villa, Mumbai, India) was dissolved in deionized (DI) water by stirring at a constant temperature of 80 °C for 4 h. Sodium alginate (SA) (alginic acid sodium salt (medium viscosity), Sigma-Aldrich, St. Louis, MO, USA) was similarly dissolved in DI water by stirring at a constant temperature of 25 °C for 2 h. After homogenous solutions were achieved, PVA and SA were mixed at weight ratios of 1:1, 1:2, 1:4, and 1:6 (SA:PVA) at 25 °C to obtain the SA/PVA solution. Next, silk sericin (SS) (sericin was extracted from a *Bombyx mori* cocoon [27]) was added to the well-stirred SA/PVA solution. Subsequently, curcumin (Cur) (10 mg/mL) (28260, Fluka, Steinheim, Germany) in an ethanol solution was added, and the mixture was stirred at a constant temperature of 25 °C for 1 h to ensure complete dissolution of SS and Cur in the SA/PVA solution. The final solution was then dropped onto a polypropylene plate (8.5 × 8.5 cm^2^, 15 g/plate) and dried uniformly at 40 °C for 24 h.

#### 2.1.2. Calcium Chloride-Crosslinked SS–Cur-Loaded SA/PVA Films

The resulting films were then crosslinked using a 0.1 M calcium chloride solution (Qrec, Auckland, New Zealand) mixed with ethanol (99.9%, Qrec, New Zealand) at various concentrations (0, 10, 20, and 30%). The crosslinking solution was poured over the dry film mold and soaked at 25 °C for 1 h. This process allowed the calcium chloride and ethanol mixture to crosslink with the film. After soaking, the residual calcium chloride solution was decanted and replaced with reverse osmosis (RO) water. This step was repeated three times for 15 min each to remove excess calcium chloride. The films were then dried at 40 °C for 24 h to obtain calcium chloride-crosslinked SS–Cur-loaded SA/PVA films. The formulations of these crosslinked films were prepared according to the ratios outlined in Table 1.

### 2.2. Characterization of SS–Cur-Loaded SA/PVA Films

#### 2.2.1. Gel Fraction

Analysis of the gel fraction percentage of the modified films was conducted according to a previous study [28]. Film samples measuring 1 × 1 cm^2^ were dried in an oven (Memmert brand, model BE. 400, Memmert Company, Schwabach, Germany) at 50 °C for 24 h. The dried samples were then soaked in DI water at 37 °C for 72 h. After soaking, the samples were removed from the water and dried again in the oven at 50 °C for another 24 h. The gel fraction percentage was calculated using the following equation:

$$\%Gel\;fraction=\frac{Weight\;of\;dried\;sample\;\mathrm{after}\;soaking}{Initial\;weight\;of\;dried\;sample}\times 100$$

#### 2.2.2. Swelling Properties

The swelling properties of the SS–Cur-loaded SA/PVA films were determined by calculating the difference between the dry weight and the wet weight of the film samples. Initially, the dried film samples were weighed and then immersed in Dulbecco’s Modified Eagle Medium (DMEM) solution at 37 °C for 24 h. After immersion, the swollen samples were carefully removed, blotted with filter paper to remove excess DMEM, and immediately weighed. The swelling percentage was calculated using the following equation:

$$\%Swelling=\frac{Weight\;of\;swollen\;sample-Initial\;weight\;of\;dried\;sample}{Initial\;weight\;of\;dried\;sample}\times 100$$

#### 2.2.3. Scanning Electron Microscopy (SEM)

The morphology and thickness of the SS–Cur-loaded SA/PVA films were characterized using scanning electron microscopy (SEM-JSM-IT500HR, JEOL, Tokyo, Japan). The thickness measurements were analyzed with ImageJ software version 1.4.3.

#### 2.2.4. Attenuated Total Reflectance Fourier Transform Infrared Spectroscopy (ATR-FTIR)

ATR-FTIR spectra were recorded using a Fourier Transform Infrared Spectrometer (INVENIO S, Bruker, Ettlingen, Germany). The spectra were captured between 400 and 4000 cm^−1^ with a spectral resolution of 4 cm^−1^. Data were generated based on the acquired spectra and presented as absorbance-versus-frequency plots (Y–X plots). Measurements were performed at room temperature.

#### 2.2.5. Thermal Properties

The thermogravimetric properties of the films were studied using a Simultaneous Thermal Analyzer (STA) (Netzsch STA 449F3, Selb, Germany). Samples weighing between 4 and 6 mg were placed in aluminum pans and tested under a nitrogen atmosphere. Measurements were conducted from 35 °C to 800 °C at a constant heating rate of 10 °C/min.

#### 2.2.6. Determination of Drug Content in SA/PVA Films

The drug content in the SA/PVA films was determined by placing film samples (1 × 1 cm^2^) in 1 mL of a PBS buffer solution (pH 7.4) and incubating them in a shaking water bath (Lab Tech brand, model LSB-030S, Daihan Labtechasia PTE, Gyeonggi-do, Republic of Korea) at 37 °C and 100 rpm for 24 h. After this incubation period, 1 mL of each sample was collected to determine the amount of drug and replaced with 1 mL of a fresh PBS solution. The films were then incubated at 80 °C for 2 h to completely dissolve the film. The amount of Cur was analyzed by UV spectrophotometry at 430 nm, using a standard curve of curcumin for calibration. The SS protein content was measured with a BCA protein assay kit (Pierce BCA Protein Assay kit 23225 23227, Thermo Scientific, Rockford, IL, USA) at an absorbance value of 562 nm, compared to a standard curve. The drug content in the films was calculated using the following equation:

$$Drug\;loading\;in\;film=\frac{Total\;amount\;of\;measurable\;drug\;(\mu g)\;\;}{Weight\;of\;initial\;dry\;film\;(mg)}\times 1000$$

#### 2.2.7. Estimation of Curcumin and Sericin Release In Vitro

To quantify the amount of drug released from the films, samples (1 × 1 cm^2^) were placed in 1 mL of Tris buffer in 2% tween 20 and 4% ethanol (pH 7.4) and incubated in a shaking water bath (Lab Tech brand, model LSB-030S, Daihan Labtechasia PTE, Namyangju, Republic of Korea) at 37 °C and 100 rpm for 0–72 h. At specified time intervals, 1 mL aliquots of the sample were removed, and an equal volume (1 mL) of fresh Tris buffer was replaced to maintain a constant volume of dissolution medium. The amount of curcumin and sericin released was analyzed using a UV spectrophotometer at wavelengths of 430 nm and 562 nm, respectively, compared to standard curves.

### 2.3. Biological Properties

#### 2.3.1. Cell Culture

L929 mouse fibroblasts were grown in Dulbecco’s Modified Eagle’s Medium (DMEM) supplemented with 10% (*v*/*v*) fetal bovine serum (Gibco, Paisley, UK) and 1% gentamycin (Gibco, Grand Island, NY, USA). The cells were maintained in an atmosphere of 5% CO_2_ at 37 °C.

#### 2.3.2. Cell Cytotoxicity Test

The effect of the films on cell viability was measured using the methylthiazolyldiphenyl–tetrazolium bromide (MTT) assay. Films were incubated in serum-free media at 10 mg/mL for 24 h. This extract, containing the leach-out products from the membrane, was filtered through a 0.22-μm filter and used for cell viability testing. L929 cells were seeded in a 96-well plate at a density of 1 × 10^4^ cells/well and incubated for 24 h at 37 °C in a 5% CO_2_ humidified atmosphere. After incubation, the medium in each well was replaced with 100 μL of varying treatments for 24 h. Following treatment, the MTT assay was performed according to standard protocols, and the absorbance at 570 nm was read on a microplate reader (Varioskan, Thermo Fisher, Waltham, MA, USA).

#### 2.3.3. Intracellular Antioxidant Activity

L929 cells were seeded in a 96-well plate at a density of 1 × 10^4^ cells/well and incubated for 24 h with various treatments of film extracts. Subsequently, the cells were treated with DCFDA (20 μM), a common ROS marker, at 37 °C for 30 min in the dark. After washing the cells with a 1X PBS solution, they were treated with H_2_O_2_ (0.8 mM) for 1 h. To detect ROS production, fluorescence intensity (excitation at 485 nm and emission at 527 nm) was measured using a microplate reader (Varioskan, Thermo Fisher, Waltham, MA, USA).

### 2.4. Statistical Analysis

All analyses were performed in triplicate to determine mean values and standard deviations (SD) using GraphPad Prism 9 software. Significant differences among multiple groups were assessed using one-way ANOVA, with a *p*-value < 0.05 considered statistically significant.

## 3. Results

### 3.1. Physical Properties of the Films

**Figure 1:**
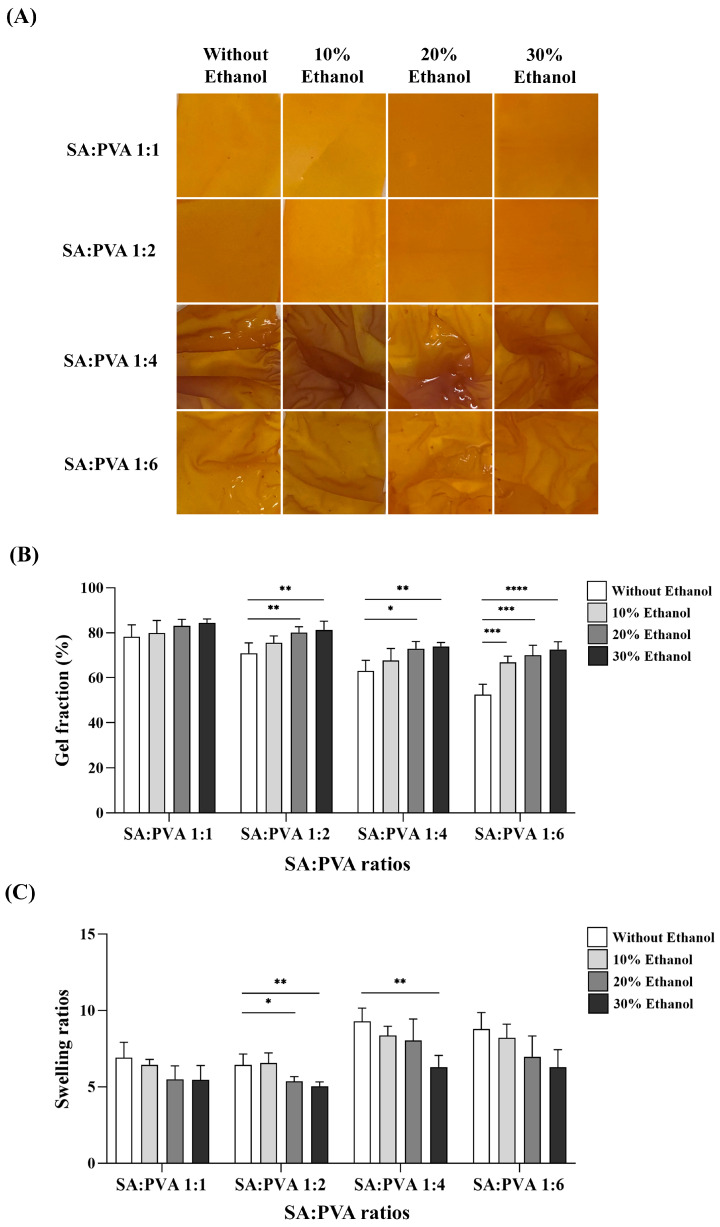
Physical properties of SS–Cur-loaded SA/PVA films crosslinked with calcium chloride: (**A**) visual appearance, (**B**) gel fraction, and (**C**) gel swelling ratio of SS–Cur-loaded SA/PVA films at different SA:PVA ratios crosslinked with calcium chloride at different ethanol concentrations (0, 10, 20, and 30%). Data are presented as the mean ± S.D (*n* = 4). * *p* ≤ 0.05, ** *p* ≤ 0.01, *** *p* ≤ 0.001, **** *p* ≤ 0.0001.

Figure 1A shows an optical image of the SS–Cur-loaded SA/PVA films after crosslinking with calcium chloride at various ratios. The preparation of SS–Cur-loaded films from SA, PVA, and the addition of ethanol in calcium chloride showed that increasing the PVA ratio and ethanol concentrations significantly affected gel formation. The gel fractions of the SS–Cur-loaded SA/PVA films at different ratios are confirmed in Figure 1B. Films with SA:PVA ratios of 1:4 and 1:6 crosslinked with calcium chloride exhibited substantial swelling and lacked the stability of a smooth sheet, whereas films with SA:PVA ratios of 1:1 and 1:2 achieved the desired gel fraction value. As shown in Figure 1C, films with SA:PVA ratios of 1:1 and 1:2 presented a lower water swelling ratio compared to films with a higher PVA content. Additionally, crosslinking with calcium chloride in the presence of increased ethanol concentrations resulted in a decreased percentage of water swelling, possibly due to the enhanced stability of the film structure.

### 3.2. Morphology and Thickness of the SS–Cur-Loaded SA/PVA Films

**Figure 2:**
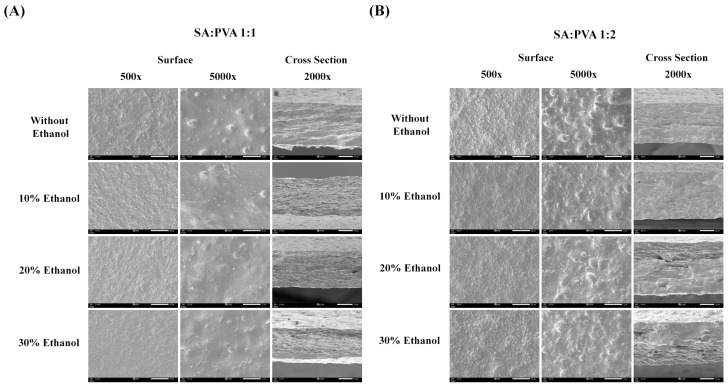
SEM images of SS–Cur-loaded SA/PVA films crosslinked with calcium chloride at SA:PVA ratios of (**A**) 1:1 and (**B**) 1:2, crosslinked with calcium chloride at different ethanol concentrations (0, 10, 20, and 30%).

Figure 2 illustrates the structural images of SS–Cur-loaded SA/PVA films with SA:PVA ratios of 1:1 and 1:2. The surface of the SA:PVA 1:2 films appears rougher than that of the 1:1 films. The thickness of the films can be adjusted by varying the SA:PVA ratio and the concentration of ethanol used during crosslinking. The addition of ethanol to the calcium chloride solution significantly affects the film thickness. The results indicate that the film thickness of SA:PVA 1:1 crosslinked with 10% ethanol in calcium chloride was the greatest. However, as the ethanol concentration increased to 20–30%, the film thickness decreased. By contrast, the SA:PVA 1:2 ratio crosslinked with 20% ethanol presented the thickest films. A summary of the film thickness measurements is provided in Table 2. These findings highlight the influence of both the SA:PVA ratio and the ethanol concentration on the morphological characteristics and thickness of the films.

### 3.3. FTIR Analysis of SS–Cur-Loaded SA/PVA Films Crosslinked with Calcium Chloride

**Figure 3:**
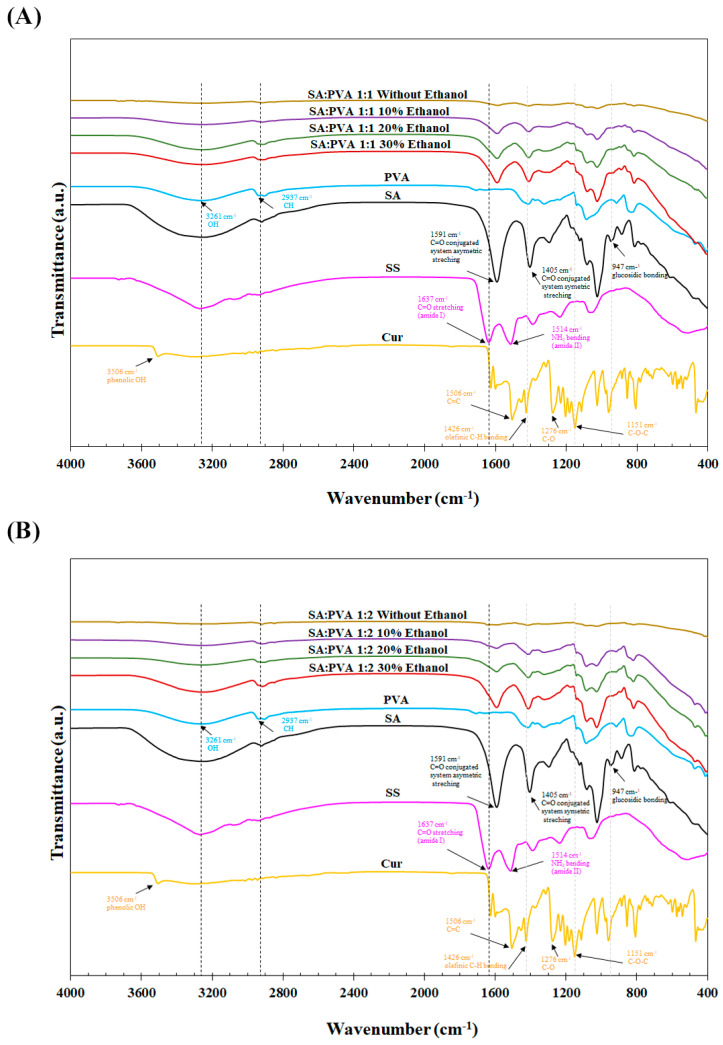
FTIR spectra of SS–Cur-loaded SA/PVA films crosslinked with calcium chloride at SA:PVA ratios of (**A**) 1:1 and (**B**) 1:2, crosslinked with calcium chloride at different ethanol concentrations (0, 10, 20, and 30%).

The chemical properties of the SS–Cur-loaded SA/PVA films crosslinked with calcium chloride at different ratios were analyzed using ATR-FTIR, as shown in Figure 3. Both the SA:PVA 1:1 and 1:2 films crosslinked with calcium chloride exhibited peaks at 1080 cm⁻^1^, indicative of the crosslinking of alginate. Crosslinking with calcium chloride at various ethanol concentrations resulted in an intense G block peak at 932 cm^−1^. Additionally, an amide I band of sericin appeared in all formulations, attributed to the C=O peaks of alginate, which affect the combination of signals. The characteristic peaks of Cur at 1426 cm⁻^1^ and 1151 cm⁻^1^ were also present in the films. These FTIR results confirm the successful incorporation and interaction of SS and Cur within the SA/PVA films.

### 3.4. Thermal Properties of SS–Cur-Loaded SA/PVA Films Crosslinked with Calcium Chloride

**Figure 4:**
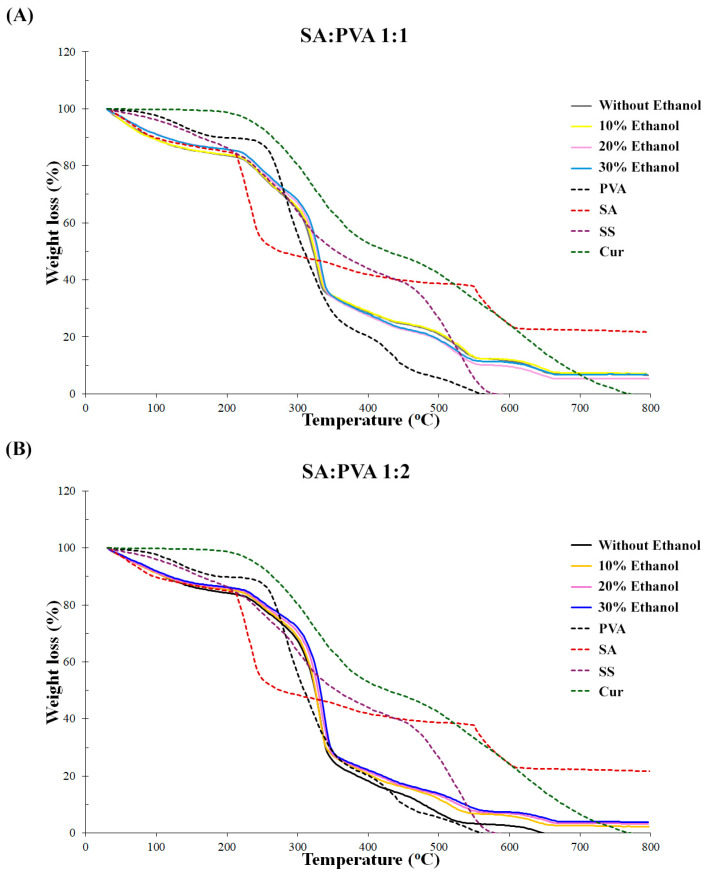
Thermal degradation curves of SS–Cur-loaded SA/PVA films crosslinked with calcium chloride at SA:PVA ratios of (**A**) 1:1 and (**B**) 1:2, crosslinked with calcium chloride at different ethanol concentrations (0, 10, 20, and 30%).

The thermal properties of SS–Cur-loaded SA/PVA films crosslinked with calcium chloride at various ratios were investigated using thermogravimetric analysis (TGA) and a Simultaneous Thermal Analyzer (STA). The thermodynamic stability of the modified films is illustrated in Figure 4. The results indicated that SA:PVA 1:1 films crosslinked with different ethanol concentrations exhibited similar weight loss profiles across formulations. In contrast, the thermal stabilities of the films improved significantly for the SA:PVA 1:2 ratio with the addition of ethanol. The SA:PVA 1:2 films crosslinked with calcium chloride without ethanol solutions experienced the highest rate of degradation. However, the films crosslinked with calcium chloride in ethanol solutions (10%, 20%, and 30%) displayed similar thermal degradation patterns, demonstrating enhanced stability. The multiple decomposition stages observed between 150 and 350 °C (attributed to SA, PVA, SS, and Cur) confirmed the presence and interaction of SS and Cur within the SA/PVA films. These findings highlight the beneficial impact of ethanol in the crosslinking process, contributing to the improved thermal stability of the sericin–curcumin-loaded SA/PVA films.

### 3.5. Drug Content and Release Profile of SS and Cur

**Figure 5:**
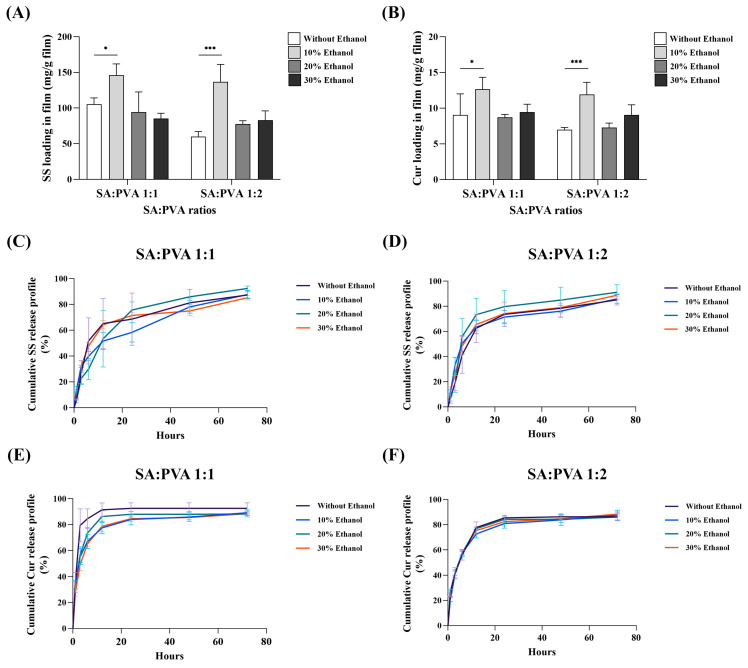
Drug loading capacity and in vitro drug release profiles of SS–Cur-loaded SA/PVA films crosslinked with calcium chloride: drug content of (**A**) SS and (**B**) Cur in SA/PVA films crosslinked with calcium chloride and in vitro release profiles of (**C**,**D**) SS and (**E**,**F**) Cur from SA/PVA films crosslinked with calcium chloride at different ethanol concentrations (0, 10, 20, and 30%). Data are represented as mean ± SD (*n* = 4). * *p* ≤ 0.05, *** *p* ≤ 0.001.

The drug content of SS and Cur in the films was analyzed to evaluate the loading capacity of the SA/PVA films. The results indicated that incorporating 10% ethanol in the crosslinking process led to the highest loading capacity of both SS and Cur in the SA/PVA films (Figure 5A,B). In terms of drug release, the in vitro profiles revealed that increasing the ethanol concentration in the SS–Cur-loaded SA/PVA films affected the release behavior differently depending on the SA:PVA ratio. Specifically, for the SA:PVA ratio of 1:1, higher ethanol concentrations resulted in a slower release rate of Cur compared to films crosslinked without ethanol (Figure 5E). However, with an SA:PVA ratio of 1:2, the ethanol concentration had minimal impact on the Cur release rates (Figure 5F). Conversely, the release of SS from the films demonstrated that the presence of ethanol enhanced the release of SS (Figure 5C,D). However, there was no significant difference between the films with and without ethanol at 72 h.

### 3.6. Cytotoxicity Evaluation of SS–Cur-Loaded SA/PVA Films Crosslinked with Calcium Chloride

**Figure 6:**
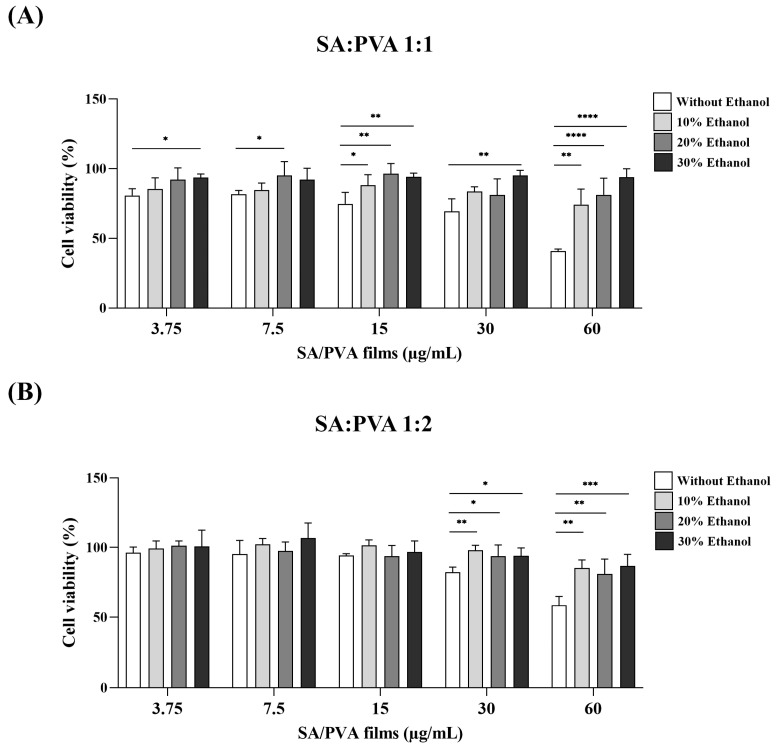
In vitro cytotoxicity of SS–Cur-loaded SA/PVA films crosslinked with calcium chloride towards L929 cells: MTT assay results showing cytotoxicity profiles of SS–Cur-loaded SA/PVA films at SA:PVA ratios of (**A**) 1:1 and (**B**) 1:2, crosslinked with calcium chloride at different ethanol concentrations (0, 10, 20, and 30%). Data are presented as mean ± S.D (*n* = 4). * *p* ≤ 0.05, ** *p* ≤ 0.01, *** *p* ≤ 0.001, **** *p* ≤ 0.0001.

The cytotoxicity of various formulations of calcium chloride-crosslinked SS–Cur-loaded SA/PVA films was assessed using L929 cells, as shown in Figure 6. SS–Cur-loaded SA/PVA films crosslinked with calcium chloride at concentrations of 3.75, 7.5, 15, 30, and 60 μg/mL were tested in L929 cells for 24 h. The results indicated that films with an SA:PVA ratio of 1:1 crosslinked with calcium chloride without ethanol exhibited higher toxicity than those with a 1:2 ratio. Additionally, films crosslinked with calcium chloride in ethanol solutions at concentrations of 10%, 20%, and 30% demonstrated reduced cytotoxicity. This indicates that the presence of ethanol in the crosslinking process mitigates the cytotoxic effects of the films.

### 3.7. Inhibition of H_2_O_2_-Induced ROS in L-929 Cells

**Figure 7:**
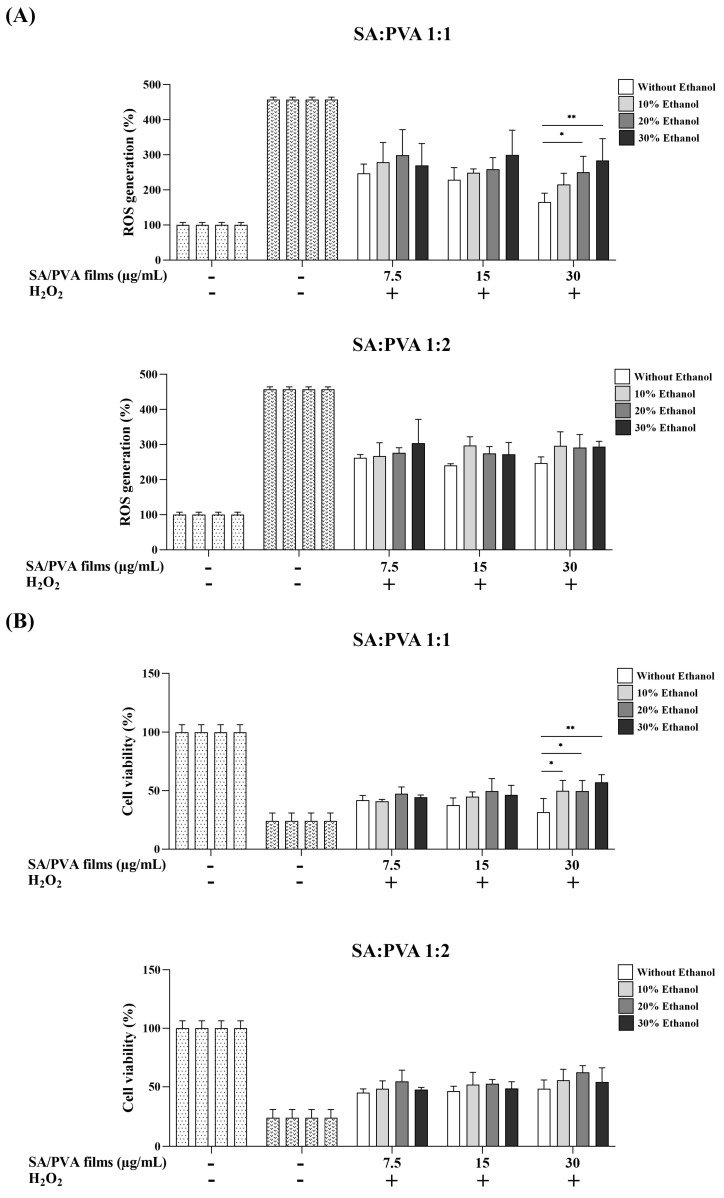
Mitigation of H_2_O_2_-induced oxidative stress by SS–Cur-loaded SA/PVA films crosslinked with calcium chloride: (**A**) intracellular ROS levels measured by DCFH-DA assay in oxidative stress-induced cytotoxicity in L929 cells treated with films at SA:PVA ratios of 1:1 and 1:2 and (**B**) cell viability assessed by MTT assay for films at SA:PVA ratios of 1:1 and 1:2, crosslinked with calcium chloride at different ethanol concentrations (0, 10, 20, and 30%). Data are presented as mean ± S.D (*n* = 4). * *p* ≤ 0.05, ** *p* ≤ 0.01.

The SS–Cur-loaded SA/PVA films crosslinked with calcium chloride demonstrated significant antioxidant activity, as illustrated in Figure 7. The SS–Cur-loaded SA/PVA films, whether crosslinked with or without ethanol, effectively reduced ROS levels, presenting their protective effect (Figure 7A). Furthermore, an increase in cell viability was observed in L-929 cells treated with SS–Cur-loaded SA/PVA films crosslinked with calcium chloride following H_2_O_2_ induction, compared to cells treated with H_2_O_2_ alone. Notably, the SA:PVA ratios of 1:1 crosslinked with calcium chloride in ethanol at 30 μg/mL were more protective than those without ethanol in preventing cell death. The MTT assay results (Figure 7B) confirmed this finding, indicating that these films crosslinked with calcium chloride in ethanol solutions effectively mitigated oxidative stress-induced damage.

## 4. Discussion

Wound dressings with excellent properties have been developed to enhance wound healing capabilities. Various natural and synthetic polymers have been utilized in their manufacture. Among these, PVA (polyvinyl alcohol) is one of the oldest and most frequently studied synthetic polymers due to its favorable wound dressing properties, such as hydrophilicity, biocompatibility, biodegradability, chemical resistance, adhesion, non-toxicity, cost-effectiveness, and high mechanical properties [29,30,31,32]. However, PVA alone has limitations in instability and is insufficient for use in wound dressings due to its lack of exudate absorption capacity, low elasticity, and inability to deliver drugs effectively [29,33,34]. To address these limitations, previous studies have developed PVA-based dressings by incorporating other polymers such as dextran, chitosan, gelatin, or hyaluronic acid [35,36,37,38]. This study focuses on combining PVA with SA (sodium alginate) to overcome these issues. SA is recognized for its excellent biocompatibility, high absorbency, and ability to form hydrogels, making it suitable for advanced wound management [39]. Additionally, SA possesses hemostatic properties and provides a moist environment conducive to wound healing. The integration of PVA with SA aims to combine the advantageous properties of both materials, resulting in an advanced wound dressing with enhanced functionality [12].

Our study fabricated a film composed of SA and PVA with ratios of 1:1, 1:2, 1:4, and 1:6 (wt/wt%). Ionic crosslinking significantly affects the physical and mechanical properties of the films. Among various ionic crosslinking agents, calcium ions (Ca^2^⁺) have been shown to provide the greatest tensile strength, highest elongation, and best light transmission in alginate film [40]. Furthermore, crosslinking alginate with Ca^2^⁺ impacts its drug release profiles [41] as well as its mechanical and biochemical properties [42]. Adding ethanol to the calcium chloride solution further improves the mechanical properties of alginate films [43]. To enhance film properties in this study, the resulting films were crosslinked with calcium chloride in 10%, 20%, and 30% ethanol solutions. Our study is the first study to explore the advantages of ethanol in a calcium chloride solution in both chemical, physical, and biological properties. The films with SA:PVA ratios of 1:4 and 1:6 crosslinked with calcium chloride, with or without ethanol, exhibited instability (Figure 1). At these higher PVA ratios, the films became too flexible and lacked sufficient structural integrity. The lower amount of alginate might not have been enough to form a stable network within the film, leading to excessive moisture absorption, swelling, and instability [44]. Optimal mechanical strength requires a balance between the flexibility provided by PVA and the rigidity provided by alginate. Moreover, our results showed that varying ethanol concentrations affected gel formation and swelling behavior (Figure 1B,C). The gel formation of SS–Cur-loaded SA/PVA films increased with higher ethanol concentrations. Ethanol facilitates ionic interaction between SA, PVA, and calcium ions, promoting the gelation process and contributing to an increase in film thickness (Figure 2). It also reduces surface tension and improves calcium ion diffusion into the SA/PVA matrix, resulting in a more uniform and robust gel formation [43,45,46]. Additionally, ethanol crosslinking affects the swelling behavior of PVA [47]. Ethanol serves as a cosolvent that can reduce the water content around calcium ions during the crosslinking process. Calcium ions (Ca^2^⁺) act as crosslinker in sodium alginate/polyvinyl alcohol (SA/PVA) gels by forming ionic bonds with the carboxyl groups on the alginate chains, creating a network that strengthens the gel. Ethanol lowers the polarity of the solution, making calcium ions more available to interact with the polymer chains rather than being surrounded by water molecules. The SS–Cur-loaded SA/PVA films in ratios of 1:1 and 1:2 showed desirable properties, leading to their use in further experiments.

FTIR analysis explored the structural and chemical interactions within SS–Cur-loaded SA/PVA films crosslinked with calcium chloride, confirming the presence of SS, Cur, and the crosslinking agents. Our result indicated the peak at the position 1080 cm^−1^, indicating the formation of calcium alginate gel [48]. Crosslinking with calcium chloride in various ethanol concentrations resulted in an intense G block at 932 cm^−1^ [49]. Peaks related to the amide I band of sericin (C=O stretching around 1600 cm⁻^1^) [50] appeared in all formulations, similar to the C=O stretching of carboxyl groups in alginate [51]. Peaks at 2900 cm⁻^1^ (C-H stretching) of PVA was observed in the SA/PVA films with increasing ethanol concentration [52]. Peaks associated with C-O-C stretching (around 1151 cm⁻^1^) indicated the presence of Cur [53]. Combined peaks from SA and PVA, with additional peaks or shifts, indicated the presence of SS and Cur (Figure 3).

Thermal stability is a crucial property that directly affects other characteristics, such as mechanical strength, durability, and shelf life [54]. The addition of various agents alters thermal stability [32]. All composite films composed of SA and PVA at a ratio of 1:1 exhibited similar thermal behavior in the TGA curves. However, the SA to PVA ratio of 1:2 without ethanol showed higher thermal degradation than all formulations containing ethanol in calcium chloride (Figure 4). These results suggest that incorporating ethanol into formulations with a higher PVA content enhances the thermal stability of SS–Cur-loaded SA/PVA films. The improved thermal performance of these films, crosslinked with calcium chloride in the presence of ethanol, can be attributed to the interactions between SA, PVA, and ethanol in calcium chloride, which create a more stable composite material. PVA’s inherent thermal stability is further supported by the presence of ethanol, which helps to form a more thermally resistant network [55]. Moreover, adding ethanol to calcium chloride improved the mechanical properties of alginate films [43]. These factors collectively improve the thermal performance of SS–Cur-loaded SA/PVA films crosslinked with calcium chloride in the presence of ethanol.

An evaluation of the drug release profiles of SS–Cur-loaded SA/PVA films revealed distinct behaviors for SS and Cur in the presence of ethanol (Figure 5). Incorporating drugs into SA/PVA films effectively controls their release due to the environmental conditions and electrostatic interactions between the drugs and the films [56,57]. In this study, electrostatic interactions play a pivotal role in controlling the release of SS and Cur from SA/PVA films. The interactions between the negatively charged SA and the positively charged calcium ions used in crosslinking create a dense polymer network, which slows the diffusion of the drug molecules, particularly Cur, by stabilizing the matrix. The different Cur release profiles in the presence or absence of ethanol were observed in films composed of SA and PVA at a 1:1 ratio. Ethanol enhances the crosslinking process, resulting in a more controlled initial release of Cur. This is likely due to the denser and more stable polymer network formed in the presence of ethanol, which slows down the diffusion of Cur. Conversely, the presence of ethanol in the calcium chloride solution did not significantly affect the SS release profile compared to formulations without ethanol. This indicates that, while ethanol plays a crucial role in modulating the release of hydrophobic compounds like Cur, its impact on the release of hydrophilic compounds such as SS is minimal. These findings highlight the selective influence of ethanol on the release kinetics of different bioactive agents in SA/PVA films.

To evaluate the potential of SS–Cur-loaded SA/PVA films crosslinked with calcium chloride as a wound dressing material, their biocompatibility and antioxidant capacity were assessed in L929 cells. SS and Cur are known for their strong antioxidant activities [58,59,60,61,62]. Antioxidants play a crucial role in wound healing by neutralizing ROS. Thus, ROS-scavenging biomaterials are promising for wound care management [20]. Many plant-derived compounds have been reported to possess both wound healing and antioxidant properties. However, their bioavailability is often limited, making it necessary to integrate antioxidants into biomaterials to maintain their activity and enhance wound management [20]. Our results indicated that ethanol-mediated crosslinking increased cell viability (Figure 6). The improved crosslinking density results in a stable polymer network, which not only controls the release of bioactive compounds but also minimizes the harmful degradation of byproducts. Interestingly, incorporating antioxidants into SA/PVA films maintained the antioxidant capacity of SS and Cur, especially in the presence of ethanol, in the H_2_O_2_-induced oxidative stress model (Figure 7). A previous study has shown that SS decreases ROS production with decreased inflammatory factors COX-2, iNOS, tumor necrosis factor-α, and interleukin-1β in H_2_O_2_-induced HaCaT cells [63]. Cur has also been shown to protect against H_2_O_2_-induced oxidative stress in human dermal fibroblasts by enhancing the expression and activities of antioxidant enzymes [58]. By neutralizing ROS, SS–Cur-loaded SA/PVA films crosslinked with calcium chloride create a more favorable environment for wound healing. However, additional benefits such as anti-inflammatory and antimicrobial effects should be further studied.

## 5. Conclusions

In conclusion, SS–Cur-loaded SA/PVA films crosslinked with calcium chloride in the presence of ethanol exhibit a synergistic combination of bioactivity, stability, and functionality, making them strong candidates for advanced wound healing applications. The addition of ethanol in the crosslinking process notably enhances the mechanical integrity and thermal stability of the films, enabling controlled, sustained release of SS and Cur. This sustained release supports ongoing antioxidant activity, which is crucial for mitigating oxidative stress and promoting a conducive environment for tissue regeneration. Furthermore, the incorporation of bioactive agents such as SS and Cur provides intrinsic therapeutic effects, enhancing biocompatibility and offering antioxidant properties essential for effective wound management. Beyond wound healing, these films show promise for broader applications, including the treatment of skin diseases. This study underscores the unique advantages of SA/PVA film matrices as versatile, biofunctional wound dressings that could significantly advance the field of skin tissue repair and care.

## Figures and Tables

**Table 1:** Composition of the chemical concentrations in the synthesis of calcium chloride-crosslinked sericin–curcumin-loaded SA/PVA films.

Ratio SA:PVA	SA (%wt)	PVA (%wt)	SS (%wt)	Cur (%wt)	Total Volume (mL)
1:1	1	1	1	0.04	50
1:2	1	2	1	0.04	50
1:4	1	4	1	0.04	50
1:6	1	6	1	0.04	50

**Table 2:** Film thickness of SS–Cur-loaded SA/PVA films crosslinked with calcium chloride.

Film Type	Film Thickness (µm)	
	SA:PVA 1:1	SA:PVA 1:2
Without Ethanol	25.83 ± 1.15	22.38 ± 1.03 #
10% Ethanol	32.24 ± 1.08 *	29.86 ± 1.88 *,#
20% Ethanol	26.63 ± 0.97	33.68 ± 2.04 *,#
30% Ethanol	22.54 ± 0.85 *	22.39 ± 1.19

* indicates statistical significance compared with and without ethanol; # indicates statistical significance between SA:PVA 1:1 and SA:PVA 1:2 at the same ethanol concentrations.

## Data Availability

The data presented in this study are available on request from the corresponding author.

## References

[1] Bakhsheshi-Rad H.R., Ismail A.F., Aziz M., Akbari M., Hadisi Z., Omidi M., Chen X. (2020). Development of the PVA/CS nanofibers containing silk protein sericin as a wound dressing: In vitro and in vivo assessment. Int. J. Biol. Macromol..

[2] Morton L.M., Phillips T.J. (2016). Wound healing and treating wounds: Differential diagnosis and evaluation of chronic wounds. J. Am. Acad. Dermatol..

[3] Kolimi P., Narala S., Nyavanandi D., Youssef A.A.A., Dudhipala N. (2022). Innovative Treatment Strategies to Accelerate Wound Healing: Trajectory and Recent Advancements. Cells.

[4] Kaur G., Narayanan G., Garg D., Sachdev A., Matai I. (2022). Biomaterials-Based Regenerative Strategies for Skin Tissue Wound Healing. ACS Appl. Bio Mater..

[5] Varaprasad K., Jayaramudu T., Kanikireddy V., Toro C., Sadiku E.R. (2020). Alginate-based composite materials for wound dressing application:A mini review. Carbohydr. Polym..

[6] Mori Y., Nakagami G., Kitamura A., Minematsu T., Kinoshita M., Suga H., Kurita M., Hayashi C., Kawasaki A., Sanada H. (2019). Effectiveness of biofilm-based wound care system on wound healing in chronic wounds. Wound Repair Regen..

[7] Zaitun Hasibuan P.A., Yuandani, Tanjung M., Gea S., Pasaribu K.M., Harahap M., Perangin-Angin Y.A., Prayoga A., Ginting J.G. (2021). Antimicrobial and antihemolytic properties of a CNF/AgNP-chitosan film: A potential wound dressing material. Heliyon.

[8] Bialik-Was K., Pluta K., Malina D., Barczewski M., Malarz K., Mrozek-Wilczkiewicz A. (2021). Advanced SA/PVA-based hydrogel matrices with prolonged release of Aloe vera as promising wound dressings. Mater. Sci. Eng. C Mater. Biol. Appl..

[9] Suhng E.A., Byun J.Y., Choi Y.W., Myung K.B., Choi H.Y. (2011). A Case of Allergic Contact Dermatitis Due to DuoDERM Extrathin(R). Ann. Dermatol..

[10] Dabiri G., Damstetter E., Phillips T. (2016). Choosing a Wound Dressing Based on Common Wound Characteristics. Adv. Wound Care.

[11] Dryden S.V., Shoemaker W.G., Kim J.H. (2013). Wound management and nutrition for optimal wound healing. Atlas. Oral Maxillofac. Surg. Clin. N. Am..

[12] Yuan N., Shao K., Huang S., Chen C. (2023). Chitosan, alginate, hyaluronic acid and other novel multifunctional hydrogel dressings for wound healing: A review. Int. J. Biol. Macromol..

[13] Narayanan R.P., Melman G., Letourneau N.J., Mendelson N.L., Melman A. (2012). Photodegradable iron(III) cross-linked alginate gels. Biomacromolecules.

[14] Saraiva M.M., Campelo M.D.S., Camara Neto J.F., Lima A.B.N., Silva G.A., Dias A., Ricardo N., Kaplan D.L., Ribeiro M. (2023). Alginate/polyvinyl alcohol films for wound healing: Advantages and challenges. J. Biomed. Mater. Res. B Appl. Biomater..

[15] Abourehab M.A.S., Rajendran R.R., Singh A., Pramanik S., Shrivastav P., Ansari M.J., Manne R., Amaral L.S., Deepak A. (2022). Alginate as a Promising Biopolymer in Drug Delivery and Wound Healing: A Review of the State-of-the-Art. Int. J. Mol. Sci..

[16] Leng Q., Li Y., Pang X., Wang B., Wu Z., Lu Y., Xiong K., Zhao L., Zhou P., Fu S. (2020). Curcumin nanoparticles incorporated in PVA/collagen composite films promote wound healing. Drug Deliv..

[17] Zhang H., Xia H., Zhao Y. (2012). Poly(vinyl alcohol) Hydrogel Can Autonomously Self-Heal. ACS Macro Lett..

[18] Pingan H., Mengjun J., Yanyan Z., Ling H. (2017). A silica/PVA adhesive hybrid material with high transparency, thermostability and mechanical strength. RSC Adv..

[19] Comino-Sanz I.M., Lopez-Franco M.D., Castro B., Pancorbo-Hidalgo P.L. (2021). The Role of Antioxidants on Wound Healing: A Review of the Current Evidence. J. Clin. Med..

[20] Joorabloo A., Liu T. (2024). Recent advances in reactive oxygen species scavenging nanomaterials for wound healing. Exploration.

[21] Dong Y., Wang Z. (2023). ROS-scavenging materials for skin wound healing: Advancements and applications. Front. Bioeng. Biotechnol..

[22] Fadilah N.I.M., Phang S.J., Kamaruzaman N., Salleh A., Zawani M., Sanyal A., Maarof M., Fauzi M.B. (2023). Antioxidant Biomaterials in Cutaneous Wound Healing and Tissue Regeneration: A Critical Review. Antioxidants.

[23] Castro B., Palomares T., Azcoitia I., Bastida F., del Olmo M., Soldevilla J.J., Alonso-Varona A. (2015). Development and preclinical evaluation of a new galactomannan-based dressing with antioxidant properties for wound healing. Histol. Histopathol..

[24] Thi P.L., Lee Y., Tran D.L., Thi T.T.H., Kang J.I., Park K.M., Park K.D. (2020). In situ forming and reactive oxygen species-scavenging gelatin hydrogels for enhancing wound healing efficacy. Acta Biomater..

[25] Kumar J.P., Mandal B.B. (2017). Antioxidant potential of mulberry and non-mulberry silk sericin and its implications in biomedicine. Free Radic. Biol. Med..

[26] Akbik D., Ghadiri M., Chrzanowski W., Rohanizadeh R. (2014). Curcumin as a wound healing agent. Life Sci..

[27] Moghadamtousi S.Z., Kadir H.A., Hassandarvish P., Tajik H., Abubakar S., Zandi K. (2014). A review on antibacterial, antiviral, and antifungal activity of curcumin. Biomed. Res. Int..

[28] Ashraaf S., Tahir H.M., Raza C., Awad E.M., Ali S., Khan S.Y., Barisani-Asenbauer T. (2023). Synergistic Effect of Silk Sericin and Curcumin to Treat an Inflammatory Condition. J. Burn Care Res..

[29] Aramwit P., Ekasit S., Yamdech R. (2015). The development of non-toxic ionic-crosslinked chitosan-based microspheres as carriers for the controlled release of silk sericin. Biomed. Microdevices.

[30] Ren T., Gan J., Zhou L., Chen H. (2020). Physically Crosslinked Hydrogels Based on Poly (Vinyl Alcohol) and Fish Gelatin for Wound Dressing Application: Fabrication and Characterization. Polymers.

[31] Jin S.G. (2022). Production and Application of Biomaterials Based on Polyvinyl alcohol (PVA) as Wound Dressing. Chem. Asian J..

[32] Kamoun E.A., Kenawy E.S., Chen X. (2017). A review on polymeric hydrogel membranes for wound dressing applications: PVA-based hydrogel dressings. J. Adv. Res..

[33] Baghaie S., Khorasani M.T., Zarrabi A., Moshtaghian J. (2017). Wound healing properties of PVA/starch/chitosan hydrogel membranes with nano Zinc oxide as antibacterial wound dressing material. J. Biomater. Sci. Polym. Ed..

[34] Voronova M.I., Surov O.V., Guseinov S.S., Barannikov V.P., Zakharov A.G. (2015). Thermal stability of polyvinyl alcohol/nanocrystalline cellulose composites. Carbohydr. Polym..

[35] Hassan A., Niazi M.B.K., Hussain A., Farrukh S., Ahmad T. (2017). Development of Anti-bacterial PVA/Starch Based Hydrogel Membrane for Wound Dressing. J. Polym. Environ..

[36] Yang W., Ding H., Qi G., Li C., Xu P., Zheng T., Zhu X., Kenny J.M., Puglia D., Ma P. (2021). Highly transparent PVA/nanolignin composite films with excellent UV shielding, antibacterial and antioxidant performance. React. Funct. Polym..

[37] Phulmogare G., Rani S., Lodhi S., Patil U.K., Sinha S., Ajazuddin, Gupta U. (2024). Fucoidan loaded PVA/Dextran blend electrospun nanofibers for the effective wound healing. Int. J. Pharm..

[38] Kim J.U., Ko J., Kim Y.S., Jung M., Jang M.H., An Y.H., Hwang N.S. (2024). Electrical Stimulating Redox Membrane Incorporated with PVA/Gelatin Nanofiber for Diabetic Wound Healing. Adv. Healthc. Mater..

[39] Zhou W., Duan Z., Zhao J., Fu R., Zhu C., Fan D. (2022). Glucose and MMP-9 dual-responsive hydrogel with temperature sensitive self-adaptive shape and controlled drug release accelerates diabetic wound healing. Bioact. Mater..

[40] Shamloo A., Aghababaie Z., Afjoul H., Jami M., Bidgoli M.R., Vossoughi M., Ramazani A., Kamyabhesari K. (2021). Fabrication and evaluation of chitosan/gelatin/PVA hydrogel incorporating honey for wound healing applications: An in vitro, in vivo study. Int. J. Pharm..

[41] Zhang M., Zhao X. (2020). Alginate hydrogel dressings for advanced wound management. Int. J. Biol. Macromol..

[42] Liling G., Di Z., Jiachao X., Xin G., Xiaoting F., Qing Z. (2016). Effects of ionic crosslinking on physical and mechanical properties of alginate mulching films. Carbohydr. Polym..

[43] Celik E., Bayram C., Akcapinar R., Turk M., Denkbas E.B. (2016). The effect of calcium chloride concentration on alginate/Fmoc-diphenylalanine hydrogel networks. Mater. Sci. Eng. C Mater. Biol. Appl..

[44] Wan L.Q., Jiang J., Arnold D.E., Guo X.E., Lu H.H., Mow V.C. (2008). Calcium Concentration Effects on the Mechanical and Biochemical Properties of Chondrocyte-Alginate Constructs. Cell. Mol. Bioeng..

[45] Li J., He J., Huang Y., Li D., Chen X. (2015). Improving surface and mechanical properties of alginate films by using ethanol as a co-solvent during external gelation. Carbohydr. Polym..

[46] Nunes M.A., Vila-Real H., Fernandes P.C., Ribeiro M.H. (2010). Immobilization of naringinase in PVA-alginate matrix using an innovative technique. Appl. Biochem. Biotechnol..

[47] Tan J., Luo Y., Guo Y., Zhou Y., Liao X., Li D., Lai X., Liu Y. (2023). Development of alginate-based hydrogels: Crosslinking strategies and biomedical applications. Int. J. Biol. Macromol..

[48] Xie L., Jiang M., Dong X., Bai X., Tong J., Zhou J. (2012). Controlled Mechanical and Swelling Properties of Poly(vinyl alcohol)/Sodium Alginate Blend Hydrogels Prepared by Freeze–Thaw Followed by Ca^2+^ Crosslinking. J. Appl. Polym. Sci..

[49] Kudo S., Otsuka E., Suzuki A. (2010). Swelling behavior of chemically crosslinked PVA gels in mixed solvents. J. Polym. Sci. Part B Polym. Phys..

[50] Badita C.R., Aranghel D., Burducea C., Mereuta P. (2020). Characterization of sodium alginate based films. Rom. J. Phys..

[51] Silva T.L.d., Vidart J.M.M., Silva M.G.C.d., Gimenes M.L., Vieira M.G.A. (2017). Alginate and Sericin: Environmental and Pharmaceutical Applications. Biological Activities and Application of Marine Polysaccharides.

[52] Ekasurya W., Sebastian J., Puspitasari D., Asri P.P.P., Asri L. (2023). Synthesis and Degradation Properties of Sericin/PVA Hydrogels. Gels.

[53] Xiao Q., Gu X., Tan S. (2014). Drying process of sodium alginate films studied by two-dimensional correlation ATR-FTIR spectroscopy. Food Chem..

[54] Yang H., Xu S., Jiang L., Dan Y. (2011). Thermal Decomposition Behavior of Poly (Vinyl Alcohol) with Different Hydroxyl Content. J. Macromol. Sci. Part B.

[55] Faham S., Ghavami R., Golmohammadi H., Khayatian G. (2019). Spectrophotometric and visual determination of zoledronic acid by using a bacterial cell-derived nanopaper doped with curcumin. Microchim. Acta.

[56] Jia Y., Hu C., Shi P., Xu Q., Zhu W., Liu R. (2020). Effects of cellulose nanofibrils/graphene oxide hybrid nanofiller in PVA nanocomposites. Int. J. Biol. Macromol..

[57] Medina Escobar S.A., Isaza Merino C.A., Meza Meza J.M. (2015). Mechanical and thermal behavior of polyvinyl alcohol reinforced with aligned carbon nanotubes. Matéria.

[58] Nazir A., Abbas M., Kainat F., Iqbal D.N., Aslam F., Kamal A., Mohammed O.A., Zafar K., Alrashidi A.A., Alshawwa S.Z. (2024). Efficient drug delivery potential and antimicrobial activity of biocompatible hydrogels of dextrin/Na-alginate/PVA. Heliyon.

[59] Mohammadi S., Ramakrishna S., Laurent S., Shokrgozar M.A., Semnani D., Sadeghi D., Bonakdar S., Akbari M. (2019). Fabrication of Nanofibrous PVA/Alginate-Sulfate Substrates for Growth Factor Delivery. J. Biomed. Mater. Res. A.

[60] Pan T., Zhang S., Fei H., Hu Y. (2023). Curcumin Protects Human Dermal Fibroblasts Exposed to Hydrogen Peroxide by Regulating Autophagy Level and Reactive Oxygen Species Generation. J. Burn Care Res..

[61] Menon V.P., Sudheer A.R. (2007). Antioxidant and anti-inflammatory properties of curcumin. Adv. Exp. Med. Biol..

[62] Kocaadam B., Sanlier N. (2017). Curcumin, an active component of turmeric (Curcuma longa), and its effects on health. Crit. Rev. Food Sci. Nutr..

[63] Kunz R.I., Brancalhao R.M., Ribeiro L.F., Natali M.R. (2016). Silkworm Sericin: Properties and Biomedical Applications. Biomed. Res. Int..

